# High-Pressure X-ray Diffraction Study of Orthorhombic Ca_2_Zr_5_Ti_2_O_16_

**DOI:** 10.1021/acs.jpcc.2c08011

**Published:** 2023-01-19

**Authors:** Tania Garcia-Sanchez, Daniel Diaz-Anichtchenko, Alfonso Muñoz, Placida Rodriguez-Hernandez, Tomas Marqueño, Mohsin Jafar, S. Nagabhusan Achary, Frederico Alabarse, Daniel Errandonea

**Affiliations:** † Departamento de Ingeniería Eléctrica, MALTA Consolider Team, Universitat Politècnica de València, Camino de Vera s/n., Valencia, 46022, Spain; ‡ Departamento de Física Aplicada—ICMUV, MALTA Consolider Team, Universitat de Valencia, Dr. Moliner 50, Burjassot Valencia, 46100, Spain; § Departamento de Física, Instituto de Materiales y Nanotecnología, MALTA Consolider Team, Universidad de La Laguna, La Laguna, Tenerife 38205, Spain; ∥ Chemistry Division, Bhabha Atomic Research Centre, Mumbai, 400085, India; ⊥ Xpress—High pressure diffraction beamline, Elettra synchrotron, Triestre, 25032, Italy

## Abstract

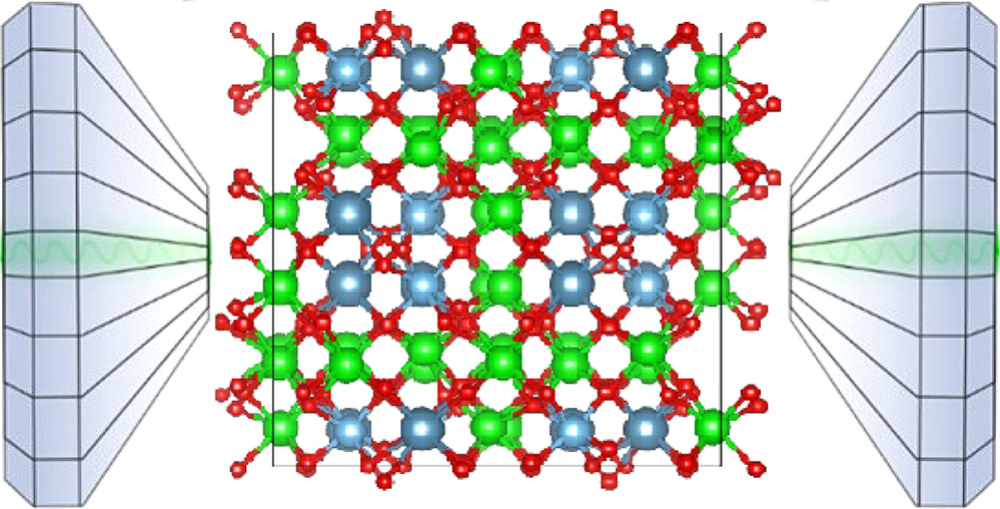

The orthorhombic polymorph of Ca_2_Zr_5_Ti_2_O_16_ (space group *Pbca*) has been studied by powder X-ray diffraction under high pressures up to 30 GPa using synchrotron radiation. We have found evidence of a structural phase transition at 12–13 GPa. The phase transition causes an enhancement of the crystal symmetry. The high-pressure phase is tetragonal, being described by space group *I*4_1_/*acd*. The space groups of the high- and low-pressure phases have a group/subgroup relationship. However, the phase transition is accompanied by a discontinuous change in the unit-cell volume, indicating that the phase transition can be classified as first order. We have also performed density functional theory calculations. These simulations support the occurrence of the orthorhombic-to-tetragonal transition. The pressure–volume equation of state and axial compressibilities have been determined for both polymorphs. The results are compared with previous studies in related oxides.

## 1 Introduction

The final disposal of complex high-level waste from nuclear reactors is one of the most important technical challenges faced by modern nations today. For instance, the immobilization of long-lived radioisotopes of radioactive waste, like actinides and lanthanides, within a highly durable host matrix is a crucial challenge for nuclear power technology.^1^ Currently, worldwide, borosilicates have been adopted for constructing interim storage immobilization matrixes for radioactive waste. However, the inherent metastable nature of glasses is a concern for using them for disposal in deep geological repositories. Additionally, the low solubilities of lanthanides and actinides and possible devitrification due to intrinsic heat generated from the stored radioactivity are unfavorable aspects of glass matrixes.^2,3^ For the purpose of nuclear waste storage, ceramics matrixes are more promising and durable as they can fix the constituent elements in the structural building frame. Different types of ceramic materials are being investigated thoroughly for this purpose. Mineral rocks and synthetic rock analogous ceramics provide guidelines for building ceramic matrixes for the immobilization of long radioactive radionuclides.^3^

Synroc, a synthetic rock composed of titanate minerals, pyrochlore, hollandite, zirconolite, and perovskite, plus rutile and a small amount of a metal alloy, was proposed in the 1970s as a cost-effective and low-risk solution for radioactive waste immobilization.^4^ The constituent minerals of synroc and their close structural analogues are capable of simultaneously immobilizing a large variety of ions in a wide range of geological environments and for long geological periods of time. These are ceramic composites of titanates and zirconates of various structure types, namely zirconolite, hollandite, pyrochlore, perovskite, and other fluorite-related oxides.^4^ A key factor for a material to be used for nuclear waste storage it is its stability under external thermodynamic conditions like pressure and/or temperature as well as in geochemical environments. It must be noted that hot isostatic pressing (HIP) technologies are already commercially employed in order to compress the mixtures of synroc with radioactive waste in order to reduce waste volumes. It is, therefore, of tantamount importance to investigate the structural and chemical stabilities of all these minerals under diversified external variables, like high pressure (HP).

**Figure 1:**
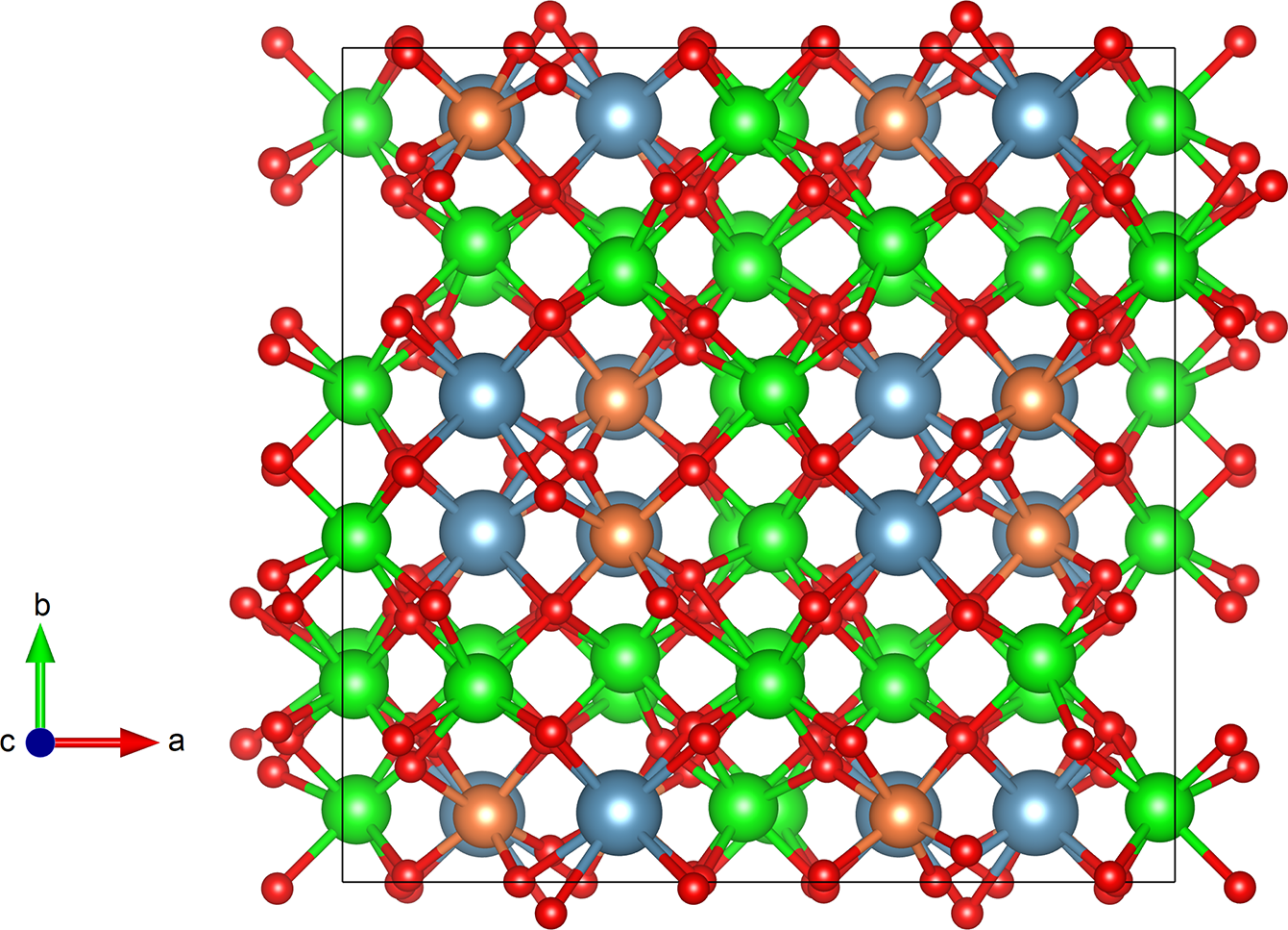
Schematic representation of the orthorhombic structure of Ca_2_Zr_5_Ti_2_O_16_. Ca, Zr, Ti, and O atoms are shown in blue, green, orange, and red, respectively. The coordination polyhedra are shown.

As the major components of synroc are based on the CaO–ZrO_2_–TiO_2_ system, extensive studies as a function of composition and temperature have been carried out in this system.^5,6^ Zirconolite (CaZrTi_2_O_7_) is one of the main components of this system and also is an important constituent of synroc.^4^ It is capable of incorporating long-lived actinide elements such as plutonium, being able to hold up to 30% of high-level waste in liquid form. Systematic studies of zirconolite as a function of composition, temperature, and pressure have been reported in the literature.^7,8^ Two important forms of this mineral are zirconolite-3T (space group *P*312) and zirconolite-2M (*C*2/*c*). The mineral calzirtite (Ca_2_Zr_5_Ti_2_O_16_) is an alternative to zirconolite.^5^ It occurs as a characteristic accessory mineral in ultramafic complexes associated with carbonatites. Its synthetic counterpart can be synthesized in the laboratory by means of solid-state reactions. Two different polymorphs are known: an orthorhombic phase stable at ambient conditions (*Pbca*)^9^ and a tetragonal high-temperature phase (*I*4_1_/*acd*).^10^ Both polymorphs are very similar and have a fluorite-related superstructure arrangement. The orthorhombic structure is represented in Figure 1. In the figure it can be seen that it consists of a network CaO_8_, TiO_6_, and ZrO_7_ polyhedra which are linked by sharing edges to form the three-dimensional framework. A comprehensive description of the crystal structure of calzirtite polymorphs can be found in the literature.^10^ One of the most interesting properties of calzirtite is its capacity to host radioactive elements by elevated concentrations, for instance, up to 17.9 wt % UO_2_ and 20.0 wt % ThO_2_.^11^ Thus, it has been proposed as a potential alternative for nuclear waste immobilization containers^11^ and, in particular, for the immobilization of stockpiled plutonium.^12^

As an external parameter, pressure has a significant effect on the structures and physical properties of materials causing phase transitions, disordering, and amorphization, among other structural changes.^13,14^ The understanding of the HP properties of zirconolite and calzirtite is very important to validate their possible use in nuclear waste repositories. In particular, HP studies are highly relevant for their security assessment in terms of mechanical stability and also in relation to the HIP processes used during nuclear waste processing. Just as an example, the occurrence of phase transitions at 5 GPa in fluorite-type Ce_2_Zr_2_O_8_^15^ excluded the use of this material for nuclear waste storage, showing the importance of high-pressure research. Although there have been reports on the high-temperature behavior of calzirtite,^10^ there is no information available on its stability under HP, which is crucial not only to understanding the fundamental behavior of the material but also to understanding its compression behavior during HIP. Note that pressure may also affect compounds like zirconolite and calzirtite in relation to geological processes like burial, gas generation, or fluid circulation. In the case of zirconolite, there is available information on the HP properties of the 2M polymorph.^7^ A phase transition has been detected at 15 GPa^7^ with a volume discontinuity of 3%. This suggests, according to chemical crystallography arguments, that calzirtite might also undergo phase transitions at similar pressures.^16^

Here, we will report an X-ray diffraction study of synthetic Ca_2_Zr_5_Ti_2_O_16_ under HP at room temperature. The aim of the study was exploring its compression behavior and determining the structural stability. We will report the axial and volume compressibilities and evidence of the occurrence of a phase transition at 12–13 GPa. No previous HP studies have been carried out on calzirtite. The results reported are relevant for technological applications and shed light on the compression behavior of an important family of fluorite-related complex oxides.

## 2 Experimental Details

Polycrystalline calzirtite (Ca_2_Zr_5_Ti_2_O_16_) was synthesized by a conventional solid-state reaction of CaCO_3_ (Sigma-Aldrich, purity 99.9%), ZrO_2_ (Sigma-Aldrich, purity 99.0%), and TiO_2_ (Sigma-Aldrich, purity 99.5%). CaCO_3_ was first dried at 473 K for 4 h, while ZrO_2_ and TiO_2_ were heated overnight at 1173 K. The mixture of CaCO_3_, ZrO_2_, and TiO_2_ in the 2:5:2 molar ratio was homogenized immersed in acetone (99.9% UPS grade). The obtained product was then pressed into pellets and heated at 1473 K for 24 h. After this heat treatment, the sample was cooled to room temperature and subsequently rehomogenized, compacted into a pellet, and again heated at 1473 K for another 24 h. Further, the sample was ground to powder. After that, the powder was compressed into pellets and sintered at 1573 K for 24 h. In all the heating and cooling cycles, the rate of heating/cooling was maintained at 2 K/min. The final product was characterized by powder X-ray diffraction. A formation of a single calzirtite phase was observed. This sample was used for high-pressure studies

A membrane-type diamond-anvil cell (DAC), with diamond culets 400 μm in diameter, was used to generate HP conditions. A stainless-steel gasket was first preindented to a 40 μm thickness, and after that a 180-μm-diameter hole was drilled, in the center of the indentation, to serve as the sample chamber. The sample was loaded together with ruby chips used to determine the pressure by means of ruby fluorescence employing the calibration provided by Dewaele et al.^17^ Neon (Ne) was used as the pressure-transmitting medium (PTM).^18^ HP-XRD measurements were performed at the Xpress beamline of Elettra synchrotron using a monochromatic wavelength of 0.4957 Å and a PILATUS 3S 6 M detector. The instrument was calibrated using CeO_2_ as standard. The two-dimensional diffraction rings obtained from the detector were integrated using Dioptas^19^ to obtain the conventional intensity versus 2θ one-dimensional diffractograms. The structural analysis was performed by employing the Rietveld technique using GSAS^20^ and Fullprof.^21^

## 3 Details of Calculations

First-principles computer simulations have proved to be a very powerful technique when combined with experimental studies of materials under high pressure.^22^ In our study, we have performed *ab initio* simulations in the framework of density functional theory (DFT) using the Vienna Ab initio Simulation Package (VASP).^23−25^ The generalized-gradient approximation (GGA) using the Armiento and Mattson (AM05), functional^26^ was used to describe the exchange–correlation energy. We have also tested the Perdew–Burke–Ernzerhof (PBE)^27^ functional and the PBE revised for solids (PBEsol)^28^ functional. However, we found that, while the optimized volume at 0 GPa agrees within 1% for AM05 calculations, it is overestimated by 2% for PBE calculations and it is underestimated by more than 2% by PBEsol calculations. On the basis of the better agreement with experiments at 0 GPa obtained with the AM05 functional, we decided to use it for HP simulations. Pseudopotentials have been used through the projector augmented wave (PAW) scheme.^29^ In the solution of the Schrödinger equation, a plane wave basis with an energy cutoff of 540 eV has been used, which assures high accuracy in the results. The integration over the Brillouin zone (BZ) has been performed with a Monkhorst–Pack^30^ grid of 2 × 2 × 2, which ensures high accuracy in the results since we are working with a primitive cell of 200 atoms. During our studies, the structural parameters of the crystalline structures and the atomic positions have been relaxed at selected volumes. During the process of relaxation and optimization of the structures, it has been required that the forces on the atoms are less than 0.003 eV/Å and the the stress tensor is diagonal with differences below 0.1 GPa, so it describes a hydrostatic situation. The enthalpy of each phase at each calculated pressure is obtained from the simulation results that give volume, energy, and pressure. By means of a second-order-polynomial fit of the enthalpy data obtained as a function of pressure, we obtain the enthalpy difference between the two phases. The mechanical and elastic properties were studied by obtaining the elastic constants using the Le Page method implemented in the VASP code.^31^

## 4 Results and Discussion

**Figure 2:**
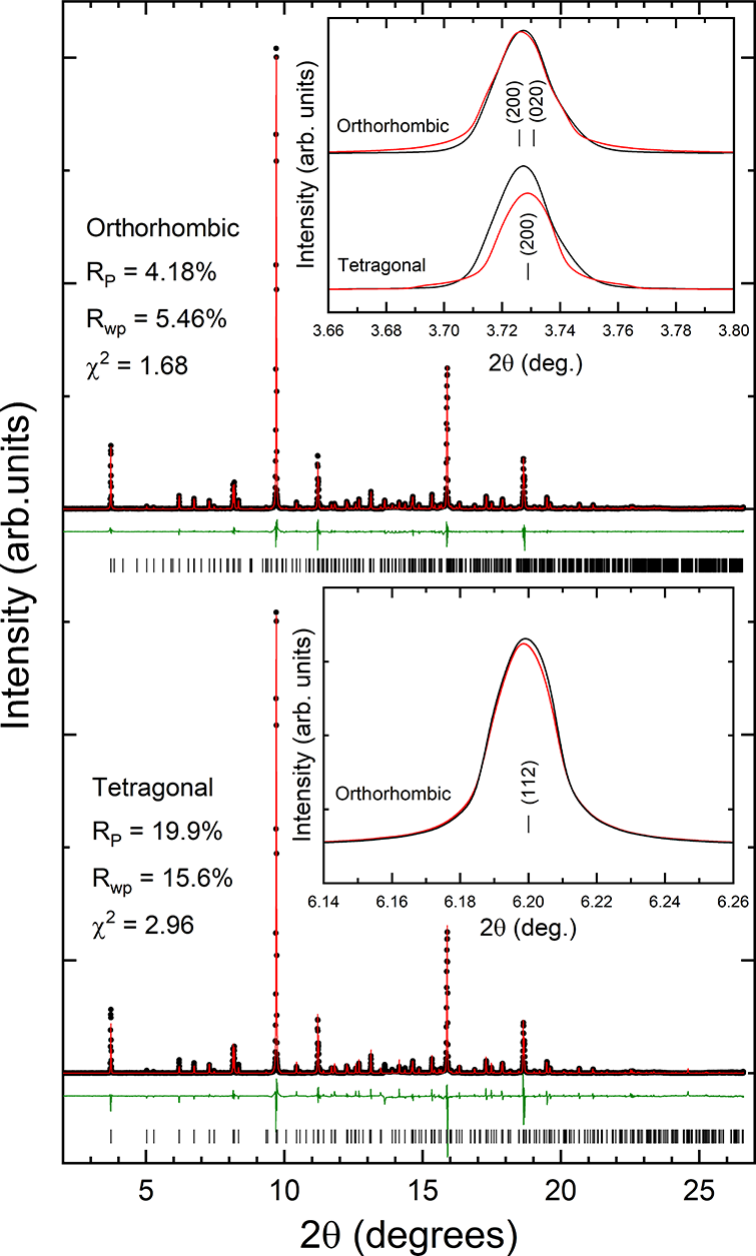
Powder XRD patterns measured in Ca_2_Zr_5_Ti_2_O_16_ at ambient pressure. The top (bottom) panel shows the Rietveld refinement using the orthorhombic (tetragonal) structure. Black dots are the experiments, red lines are the refinements, and green lines are the residuals. The ticks give the positions of calculated reflections. Inside the figure, we include the quality factors of refinements. The inset in the top panel shows a zoom of the peak at the lowest angle to highlight the better fit of the orthorhombic structure. The inset in the bottom panel shows a zoom of a peak corresponding to a single reflection to show that peak asymmetry shown in the peak of the other inset is not an experimental artifact. In both insets black lines are experiments and red lines are the fits.

The phase purity of Ca_2_Zr_5_Ti_2_O_16_ has been confirmed via a Rietveld refinement of the powder XRD patterns. All the peaks observed can be assigned to this compound. For its structure, we have considered two potential candidates: the tetragonal polymorph (space group *I*4_1_/*acd*) reported by Jafar et al.^10^ and the orthorhombic polymorph (space group *Pbca*) reported by Callegari et al.^9^Figure 2 compares the results obtained from the Rietveld refinements assuming both structures.

In Figure 2, it can be seen that the orthorhombic structure provides a better fit to the experiments than the tetragonal structure. In particular, the residuals are smaller in the orthorhombic structure and the goodness-of-fit parameters are better as shown in the figure. To illustrate that the orthorhombic structure fit the XRD pattern better than the tetragonal structure, in the inset of the top panel of Figure 2 we include a zoom of a low-angle peak. In the inset it can be clearly seen that the orthorhombic structure fits very well the low-angle peak, while the tetragonal structure cannot fit it properly. This is the peak at the lowest angle of the XRD pattern and corresponds to the (200) and (020) reflections of the orthorhombic structure. The asymmetry of the peak cannot be accounted for by the tetragonal crystal structure as shown in the inset. To show that the asymmetry of the peak is not an experimental artifact, we have also included in the bottom panel of Figure 2 a second inset showing a peak corresponding to a single reflection. It can be seen that this peak is not asymmetric. It corresponds to the (112) reflection of the orthorhombic structure. Additionally, the sample studied by Jafar et al.^10^ contains a small fraction of unreacted ZrO_2_ which was absent in the sample of the present study. The refined unit-cell parameters for the orthorhombic structure are *a* = 15.228(2) Å, *b* = 15.219(2) Å, and *c* = 10.126(2) Å, which agree with the literature.^9^ The determined atomic positions at ambient pressure are reported in Table 1.

**Table 1:** Atomic Positions in the Orthorhombic Crystal Structure of Ca_2_Zr_5_Ti_2_O_16_ at Ambient Conditions^a^

atom	site	*x*	*y*	*z*	occ
Ca1	8c	0.3320(3)	0.5813(3)	0.1267(3)	1
Ca2	8c	0.8325(3)	0.0830(3)	0.6237(3)	1
Zr1	8c	0.0137(3)	0.2628(3)	0.1255(3)	1
Zr2	8c	0.1633(3)	0.2676(3)	0.3635(3)	1
Zr3	8c	–0.0168(3)	–0.0871(3)	0.6127(3)	1
Zr4	8c	0.6605(3)	0.7681(3)	0.8575(3)	1
Zr5	8c	0.4817(3)	0.4105(3)	0.1086(3)	1
Ti1	8c	0.1713(3)	0.4210(3)	0.1269(3)	1
Ti2	8c	0.6645(3)	–0.0849(3)	0.6234(3)	1
O1	8c	0.2874(9)	–0.0005(9)	0.2521(9)	1
O2	8c	0.2495(9)	0.0374(9)	0.5005(9)	1
O3	8c	0.5699(9)	–0.0014(9)	0.2498(9)	1
O4	8c	0.2509(9)	0.3197(9)	0.5025(9)	1
O5	8c	0.2442(9)	0.3277(9)	0.2086(9)	1
O6	8c	–0.0783(9)	–0.0078(9)	0.4541(9)	1
O7	8c	0.7432(9)	0.8274(9)	0.7026(9)	1
O8	8c	0.4220(9)	0.4937(9)	–0.0448(9)	1
O9	8c	0.1110(9)	0.3316(9)	0.0033(9)	1
O10	8c	–0.0820(9)	0.8617(9)	0.2502(9)	1
O11	8c	0.6161(9)	0.8346(9)	0.4935(9)	1
O12	8c	0.4170(9)	0.3679(9)	0.7486(9)	1
O13	8c	0.0672(9)	0.3297(9)	0.4957(9)	1
O14	8c	–0.0812(9)	0.8156(9)	0.7418(9)	1
O15	8c	0.5654(9)	0.8286(9)	–0.0073(9)	1
O16	8c	0.4214(9)	0.3144(9)	0.2456(9)	1

a All atoms are at Wyckoff position 8c with an occupation (occ) equal to 1.

**Figure 3:**
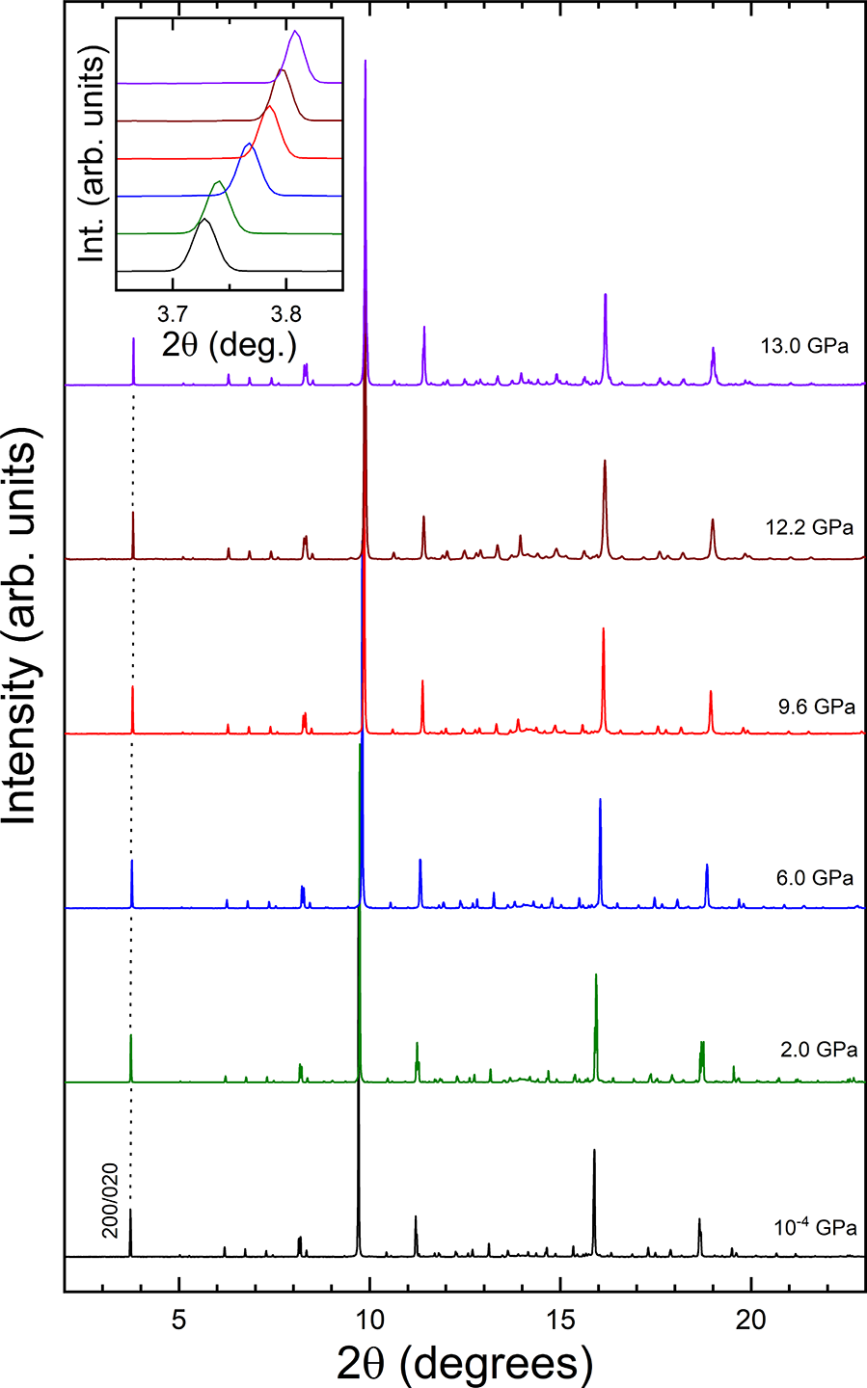
Selection of powder XRD patterns measured from ambient pressure up to 13 GPa. The inset shows a zoom of the peak corresponding to (200)/(020) reflections. The same peaks at different pressures are connected by a dashed line. The pressures are indicated in the figure. The same colors in the figure and inset correspond to the same pressures.

**Figure 4:**
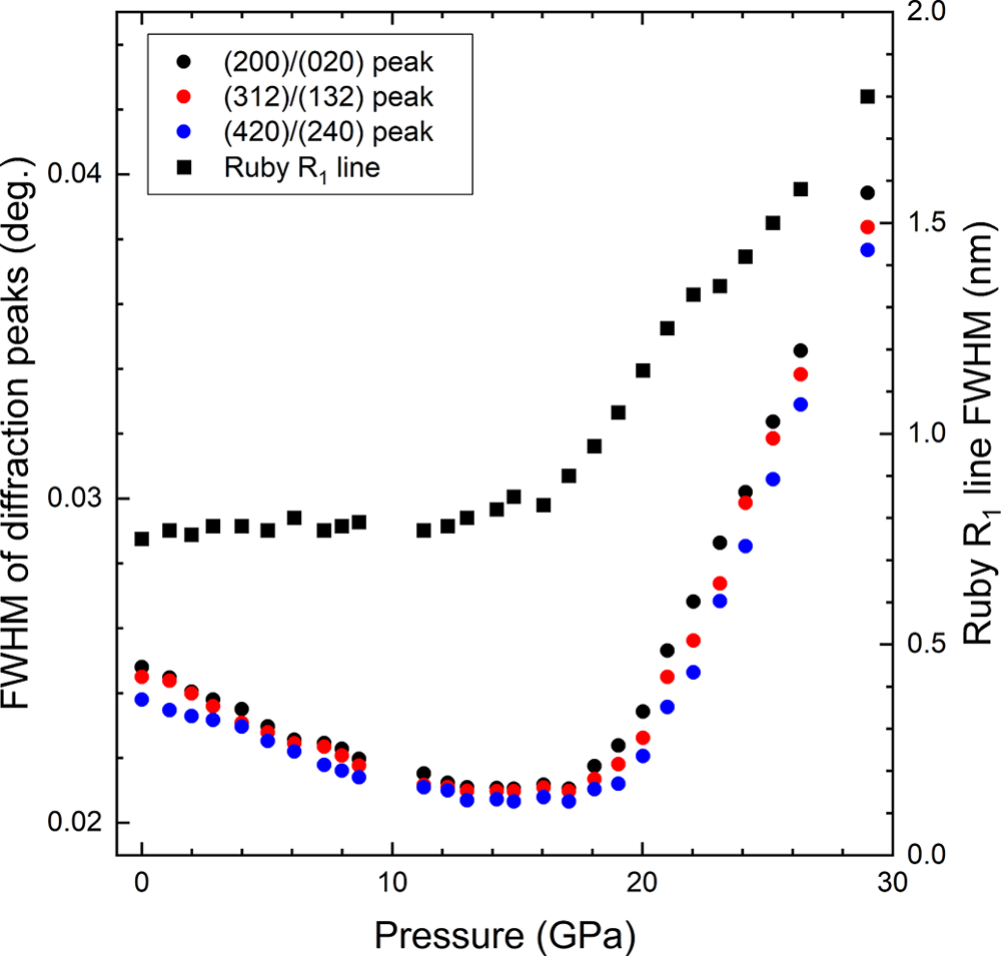
Circles represent the full width at half-maximum for three different diffraction peaks, which are identified by the Miller indexes of the orthorhombic structure (scale in the left vertical axis). Squares show the fwhm of the R_1_ line of ruby (scale in the right vertical axis).

High-pressure XRD experiments were performed up to a maximum pressure of 29 GPa. Unfortunately, we could not go to higher pressure because of a failure of the gasket containing the sample. In Figure 3 we present a selection of XRD patterns measured under compression up to 13 GPa. As the pressure increases, the peaks move gradually to higher angles due to the reduction of unit-cell parameters. In addition, we observed that the widths of several peaks decrease as pressures increases. This is illustrated in the inset of Figure 3, where it can be seen how the width of the (200)/(020) peak decreases under compression. This observation is a consequence of the fact that under compression the unit-cell parameters *a* and *b* gradually converge to the same value, which causes the merging of the two reflections. The same phenomenon has been observed for other peaks, for instance, (312)/(132) and (420)/(240) as shown in Figure 4, where we represent the full width at half-maximum (fwhm) of the three peaks as a function of pressure. The fwhm decreases up to 13 GPa for the three peaks and then remains nearly constant up to 18 GPa. Beyond this pressure it increases with pressure. This second observation will be discussed later.

**Figure 5:**
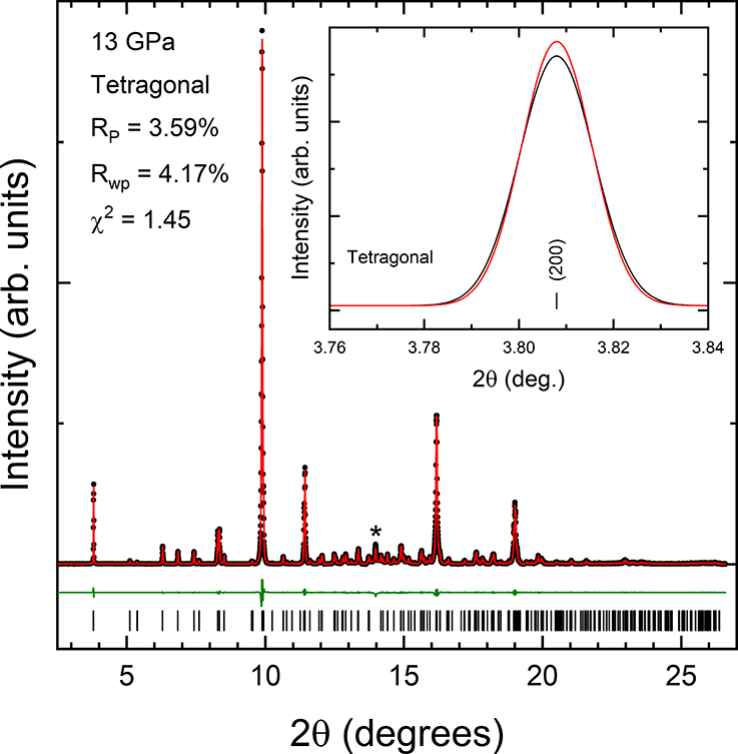
XRD pattern measured at 13 GPa showing the Rietveld refinement. Black dots are the experiments, red lines are the refinements, and green lines are the residuals. The ticks give the positions of calculated reflections. Inside the figure, we include the quality factors of refinements. The peak identified with an asterisk is a peak from solid neon. The inset shows the lowest angle peak to illustrate that at 13 GPa it can be well explained by the tetragonal structure.

The fact that the fwhm is minimum from 13 to 18 GPa added to the circumstance that at 13 GPa *a* becomes equal to *b* indicates that the symmetry of the crystal becomes tetragonal, supporting the occurrence of a phase transition. A confirmation of the phase transition comes from the fact that the XRD pattern measured at 13 GPa can be undoubtedly identified with the tetragonal structure of calzirtite (space group *I*4_1_/*a*). This is shown by the Rietveld refinement reported in Figure 5. In the inset of the figure we show a zoom of the lowest angle peak, which can be successfully explained by the (200) peak of the tetragonal structure. The refined unit-cell parameters for the tetragonal structure at 13 GPa are *a* = 14.910(2) Å and *c* = 9.924(2) Å. The determined atomic positions at 13 GPa are reported in Table 2. In the XRD pattern shown in Figure 5 there is a weak peak which could not be assigned to the sample. This peak corresponds to solid Ne, which crystallizes under compression in the fcc structure.^32^ The pressure determined from the peak, using the equation of state (EOS) reported by Dewaele et al.,^32^ agrees with that determined from the ruby scale.^17^

**Table 2:** Atomic Positions in the Tetragonal Crystal Structure of Ca_2_Zr_5_Ti_2_O_16_ at 13 GPa^a^

atom	site	*x*	*y*	*z*	occ
Ca	16f	0.3321(3)	0.3321(3)	0.25(3)	1
Zr1	16f	0.0134(3)	0.0134(3)	0.25(3)	0.5
Zr2	32g	0.1618(3)	0.0178(3)	0.4855(3)	1
Ti1	16f	0.1679(3)	0.1679(3)	0.25(3)	1
O1	16e	0.2878(9)	–0.25(9)	0.375(9)	1
O2	16e	0.5698(9)	–0.25(9)	0.375(9)	1
O3	32g	0.2432(9)	0.0778(9)	0.3303(9)	1
O4	32g	0.1138(9)	0.0824(9)	0.1240(9)	1
O5	32g	0.0656(9)	0.0796(9)	0.6195(9)	1

a The Wyckoff position (site) and occupation (occ) are also given.

**Figure 6:**
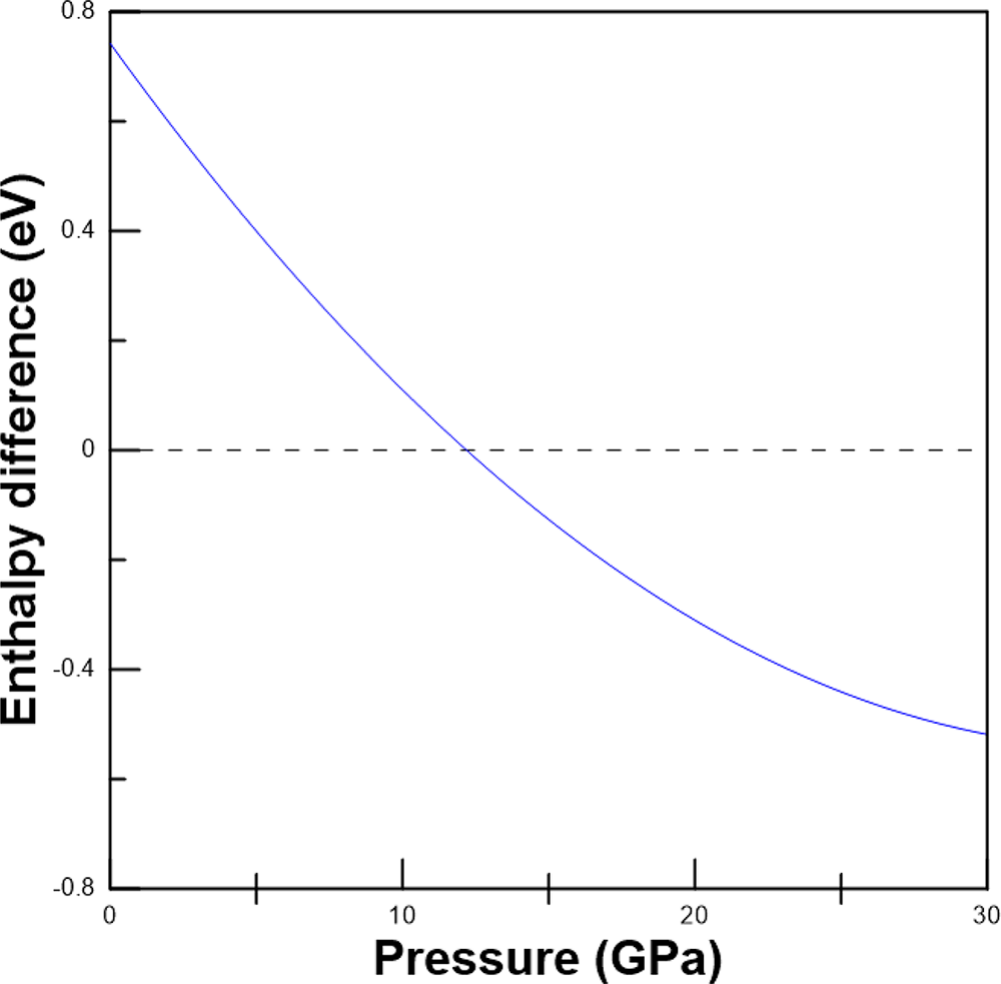
Enthalpy difference between the tetragonal (solid line) and orthorhombic (dashed line) phases using the last one as reference. The enthalpy of the tetragonal phase is the lowest beyond 12 GPa.

The observation of the orthorhombic–tetragonal transition in experiments is in full agreement with the results of present DFT calculations, which according to the minimum enthalpy criterion supports the same phase transition, giving a transition pressure of 12 GPa. Figure 6 provides the difference of enthalpy between the two polymorphs, showing that the orthorhombic phase has the minimum enthalpy up to 12 GPa and the tetragonal polymorph has the minimum enthalpy beyond 12 GPa. Thus, both calculations and experiments support that the orthorhombic phase of calzirtite is stable up to similar pressures like zirconolite.^7^

**Figure 7:**
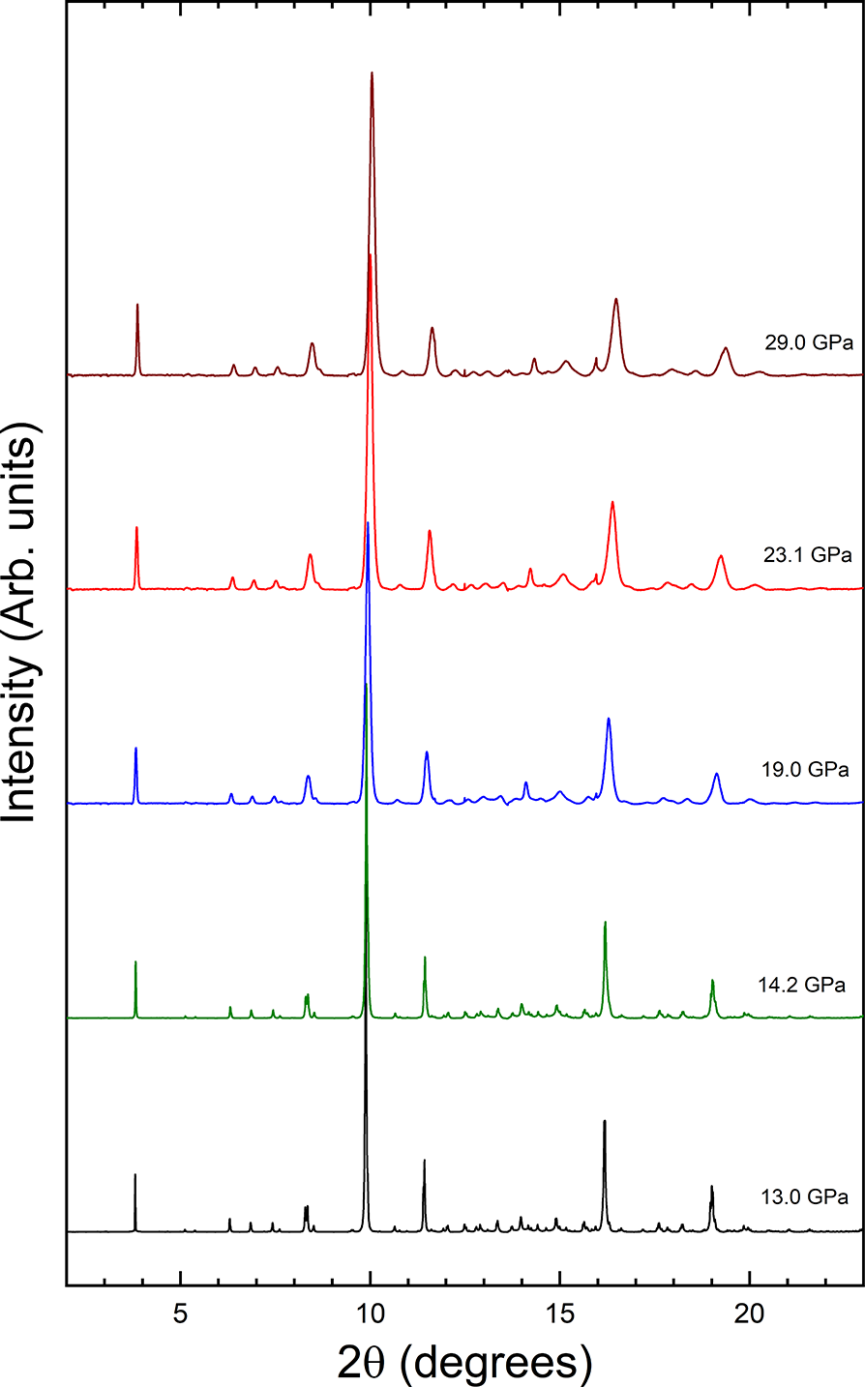
Selection of powder XRD patterns measured from 13 to 29 GPa.

Notice that a pressure of 13 GPa corresponds to a depth of approximately 410 km within the mantle of the Earth. Thus, calzirtite is suitable for building deep underground containers for storing hazardous or radioactive waste within a stable geologic environment, which are typically located at a 200–1000 m depth. Upon compression from 13 to 29 GPa, we found that calzirtite remains in the tetragonal structure. In Figure 7 we represent a selection of XRD patterns measured between the two pressures. All the patterns can be assigned to the tetragonal structure.

In Figure 7 it can be seen that at 19 GPa and higher pressures there is a broadening of the XRD peaks. This phenomenon started to develop at 18 GPa, at the same pressure where we observed that the gasket of the DAC started to shrink. Thus, we consider it is due not to a change of the crystal structure but to development of nonhydrostatic effects due to grain-to-grain stresses.^33^ The pressure at which the peak broadening begins also coincides with the pressure where neon (our pressure medium) is known to show the first signs of nonhydrostaticity.^18^ This is consistent also with the broadening we observed in the R_1_ line of ruby,^34^ which can be seen in Figure 4.

A comparison of the phase evolution of calzirtite with that of zirconolite (*C*2/*c*) shows that zirconolite transforms to a further distorted monoclinic (*P*2_1_/*m*) structure at a pressure of approximately 15 GPa, and that the HP phase remains stable up to about 56 GPa.^7^ However, in the present case the structural transition occurs with a minor reorganization of the structure which is transformed to a higher symmetry tetragonal lattice. It may be mentioned here that the orthorhombic and tetragonal structures have similar coordination polyhedra around the lattice where anions and cations are segregated into different crystallographic sites. It is also reported that the orthorhombic phase of calzirtite irreversibly transforms to the tetragonal phase at high temperature, which is due to an increasing disorder of the sites.^10^ Thus, we hypothesize that an increasing disorder in the anion sites and cation sites due to compression might be the origin of the pressure-driven orthorhombic to tetragonal transition. Further, the distinct behavior of calzirtite compared to zirconolite can be due to differences in structural arrangements. In zirconolite, the structure has a layered arrangement of cation polyhedra, and it is relatively open compared to calzirtite. In cazirtite, the structure is closely related to a fluorite-type close-packed structure, where Ca and Ti are incorporated into the lattice, making it a denser lattice. Thus, zirconolite will more likely undergo a reconstructive phase transition, but calzirtite will undergo a displacive transition from a low-symmetry to a high-symmetry form, which can be obtained by means of polyhedral tilting.

**Figure 8:**
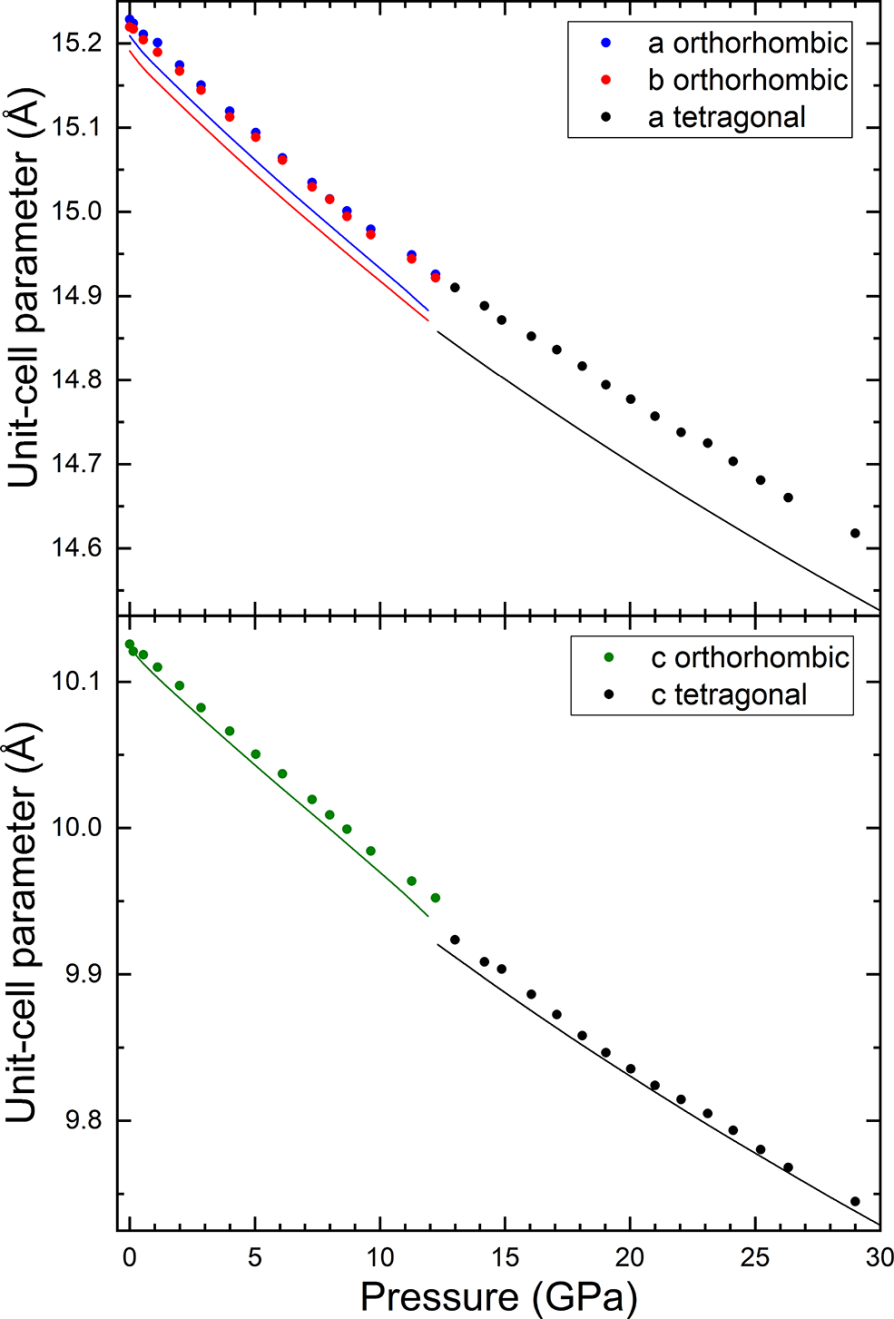
Pressure dependence of unit-cell parameters. Symbols represent the experiments, and lines represent the calculations. The same color is used for the same parameter in experimental and theoretical results.

**Figure 9:**
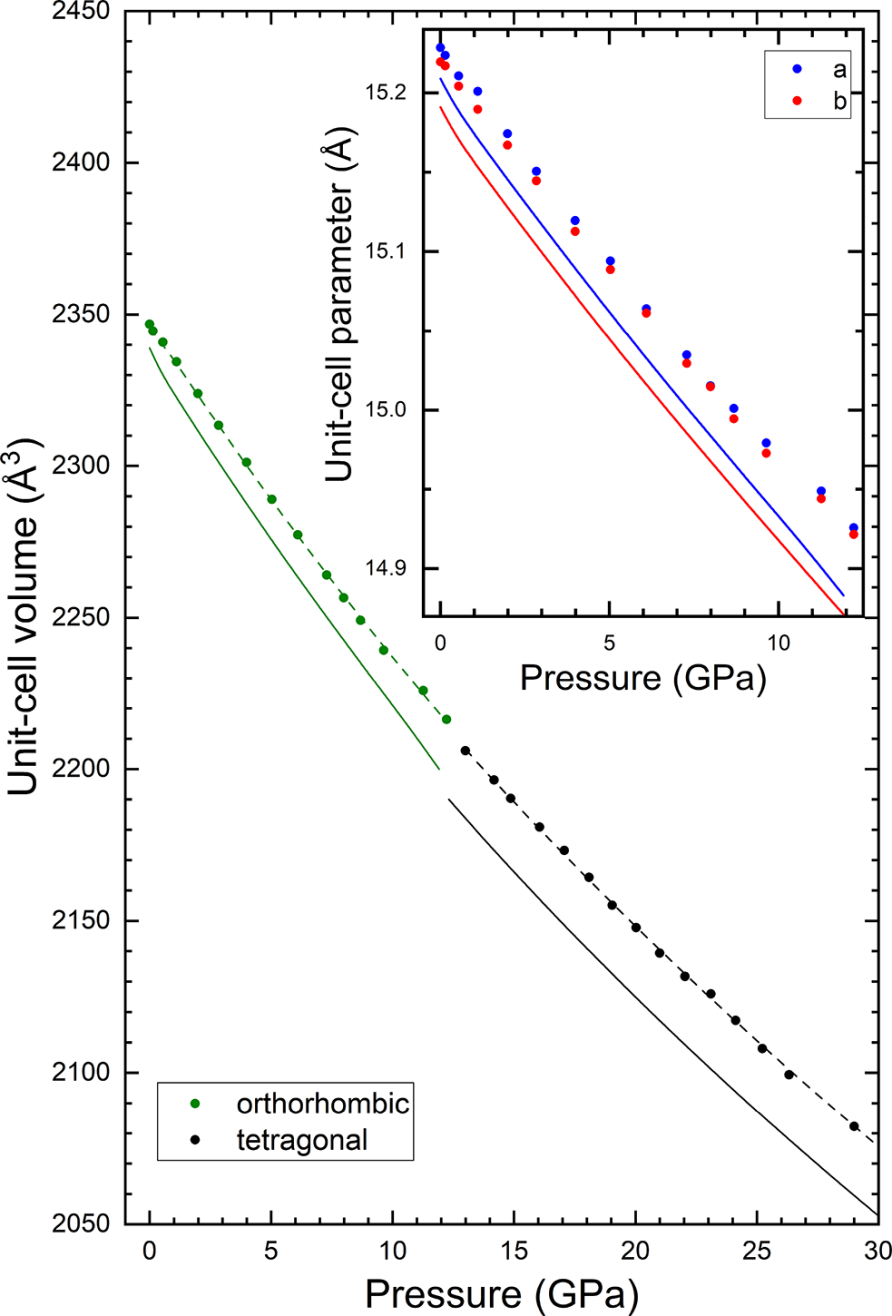
Pressure dependence of unit-cell volume. Symbols represent the experiments, solid lines represent the calculations, and dashed lines represent the equations of states determined from experiments (see EOS parameters in Tables 3 and 4). The same color is used for the same polymorph in experimental and theoretical results. The inset shows the *a* and *b* parameters for pressures up to 12.5 GPa.

From the XRD experiments, we extracted the pressure dependence of unit-cell parameters and unit-cell volume. We present the results in Figures 8 and 9. In the figures, the results from experiments are compared with results from DFT calculations. The agreement between both methods is quite good. From our results we conclude that in both phases *a*- and *b*-axes are more compressible than the *c*-axis. Interestingly, there is a discontinuity in the *c* parameter and the unit-cell volume at the phase transition. This indicates that calzirtite exhibits a first-order transition at 12–13 GPa. In the inset of Figure 9 it can be seen that *a* and *b* are different below the transition pressure.

From the results given in Figures 8 and 9, we have determined for both phases the room-temperature pressure–volume (*P*–*V*) EOS using a third-order Birch–Murnaghan (BM) EOS.^35^ We have also determined the axial linear moduli for each axis. For this purpose, we have used the formalism proposed by Angel et al. applying a modified third-order BM EOS^36^ to describe the change with pressure of unit-cell parameters. All fits have been performed using EosFit.^37^ The obtained unit-cell volume at zero pressure (*V*_0_), bulk modulus at zero pressure (*K*_0_), and its pressure derivatives (*K*_0_^′^, *K*_0_^″^) are summarized in Tables 3 and 4 for the low- and high-pressure phases, respectively. For the second derivative we give the implied values. The obtained unit-cell parameters at zero pressure (*L*_0_), the axial linear modulus at zero pressure (*M*_0_), and its pressure derivatives (*M*_0_^′^, *M*_0_^″^) are also provided in Tables 3 and 4 for both phases. For *M*_0_^″^ we report the implied value. There is a good agreement between experiments and calculations. In both phases, the response to pressure is anisotropic, with the *c*-axis being the direction of least compressibility (linear compressibilities are the inverses of the corresponding moduli).

**Table 3:** EOS Parameters for the Low-Pressure Phase of Calzirtite Determined from Experiments and Calculations^a^^,^^b^

Experiments				
	*a*-axis	*b*-axis	*c*-axis	volume
*L*_0_, *V*_0_ (Å, Å^3^)	15.230(2)	15.221(2)	10.126(1)	2347.5(5)
*M*_0_, *K*_0_ (GPa)	507(14)	528(14)	664(14)	187(4)
*M*_0_^′^, *K*_0_^′^	17.3(2.9)	14.7(2.7)	6.6(2.3)	4.2(8)
*M*_0_^″^, *K*_0_^″^ (GPa^–1^)	–0.1552	–0.0955	–0.0721	–0.0224

Calculations				
	*a*-axis	*b*-axis	*c*-axis	volume
*L*_0_, *V*_0_ (Å, Å^3^)	15.205(1)	15.188(1)	10.121(1)	2337.3(4)
*M*_0_, *K*_0_ (GPa)	504(7)	499(6)	638(11)	180(3)
*M*_0_^′^, *K*_0_^′^	10.0(1.2)	12.1(1.1)	5.0(1.7)	3.2(5)
*M*_0_^″^, *K*_0_^″^ (GPa^–1^)	–0.0655	–0.0706	–0.0980	–0.0209

a We include the unit-cell volume at zero pressure (*V*_0_) and bulk modulus at zero pressure (*K*_0_) and its pressure derivatives (*K*_0_^′^, *K*_0_^″^) as well as the unit-cell parameters at zero pressure (*L*_0_), the axial linear modulus at zero pressure (*M*_0_), and its pressure derivatives (*M*_0_^′^, *M*_0_^″^).

b Implied values are given for *M*_0_^″^ and *K*_0_^″^.

**Table 4:** EOS Parameters for the High-Pressure Phase of Calzirtite Determined from Experiments and Calculations^a^^,^^b^

Experiments			
	*a*-axis, *b*-axis	*c*-axis	volume
*L*_0_, *V*_0_ (Å, Å^3^)	15.200(5)	10.112(3)	2340(2)
*M*_0_, *K*_0_ (GPa)	527(8)	609(11)	199(4)
*M*_0_^′^, *K*_0_^′^	13.9(2.6)	13.0(2.7)	4(8)
*M*_0_^″^, *K*_0_^″^ (GPa^–1^)	–0.0841	–0.0980	–0.0224

Calculations			
	*a*-axis, *b*-axis	*c*-axis	volume
*L*_0_, *V*_0_ (Å, Å^3^)	15.159(1)	10.094(1)	2319.5(2)
*M*_0_, *K*_0_ (GPa)	543(3)	629(7)	189(3)
*M*_0_^′^, *K*_0_^′^	12.1(1.6)	13.9(1.1)	4.2(3)
*M*_0_^″^, *K*_0_^″^ (GPa^–1^)	–0.0649	–0.0980	–0.0218

a We include the unit-cell volume at zero pressure (*V*_0_) and the bulk modulus at zero pressure (*K*_0_) and its pressure derivatives (*K*_0_^′^, *K*_0_^″^) as well as the unit-cell parameters at zero pressure (*L*_0_), the axial linear modulus at zero pressure (*M*_0_), and its pressure derivatives (*M*_0_^′^, *M*_0_^″^).

b Implied values are given for *M*_0_^″^ and *K*_0_^″^.

On the other hand, the high-pressure phase has a bulk modulus 5% larger than the low-pressure phase, which is consistent with the density increase caused by the phase transition in the small discontinuity observed in the volume. For the low-pressure phase the obtained *K*_0_ and *K*_0_^′^ values are comparable to the same parameters reported for zirconolite, *K*_0_ = 188(15) GPa and *K*_0_^′^ = 3.6(1),^7^ and related compounds: Bi_2_Ti_2_O_7_, *K*_0_ = 202 GPa and *K*_0_^′^ = 2.9;^38^ Sm_2_Ti_2_O_7_, *K*_0_ = 164 GPa and *K*_0_^′^ = 4.^39^

From DFT calculations, we have also calculated the elastic constants of the low-pressure phase of calzirtite. They are summarized in Table 5. They fulfill the Born stability criteria indicating that the structure is mechanically stable. From the elastic constants we have obtained the elastic moduli and other relevant mechanical properties which are summarized in Table 6. They have been calculated using the Voigt, Reuss, and Hill approximations.^40−42^ The ratio of the bulk to shear moduli is close to 1.95, indicating that the material is ductile, with shear deformation being much easier than compression deformation. We have also obtained the elastic wave velocities which have been calculated using the equation of Navier, which are longitudinal wave velocity, 7739 m/s; transverse wave velocity, 4257 m/s; and average wave velocity, 4745 m/s.

**Table 5:** Calculated Elastic Constants of the Orthorhombic Phase of Calzirtite

elastic constant (GPa)								
*C*_11_	*C*_22_	*C*_33_	*C*_44_	*C*_55_	*C*_66_	*C*_12_	*C*_13_	*C*_23_
314.9	315.8	359.8	76.6	82.9	80.0	103.1	107.4	106.7

**Table 6:** Elastic Moduli and Other Relevant Mechanical Parameters

	Voigt	Reuss	Hill
bulk modulus (GPa)	180.5	179.8	180.2
shear modulus (GPa)	92.8	90.1	91.4
Young modulus (GPa)	237.7	231.5	234.6
Poisson ratio	0.281	0.285	0.283
P-wave modulus (GPa)	304.3	299.9	302.1
bulk/shear ratio	1.945	1.996	1.971

## 5 Conclusions

We have reported a combined experimental and theoretical study of the structural stability and mechanical properties of synthetic orthorhombic Ca_2_Zr_5_Ti_2_O_16_. By means of powder XRD measurements we determined the occurrence of an orthorhombic to tetragonal phase transition at 12–13 GPa. The structure of the high-pressure phase is that of the already known tetragonal polymorph of Ca_2_Zr_5_Ti_2_O_16_. The high-pressure phase remains stable up to 29 GPa. This observation is in full agreement with conclusions from DFT calculations. From our studies, we also determined the linear axial compressibility and pressure–volume equation of state for both polymorphs. We finally report elastic constants and moduli for orthorhombic Ca_2_Zr_5_Ti_2_O_16_. Our results indicate that orthorhombic Ca_2_Zr_5_Ti_2_O_16_ would be stable up a depth of a depth of 410 km within the mantle of the Earth. This fact and the mechanical properties of orthorhombic Ca_2_Zr_5_Ti_2_O_16_ make it a good candidate for storing nuclear waste and toxic materials.

## Data Availability

The data that support the findings of this study are available from the corresponding author upon reasonable request.
